# Antibacterial activity of Manuka honey and its components: An overview

**DOI:** 10.3934/microbiol.2018.4.655

**Published:** 2018-11-27

**Authors:** Matthew Johnston, Michael McBride, Divakar Dahiya, Richard Owusu-Apenten, Poonam Singh Nigam

**Affiliations:** 1 Biomedical Sciences Research Institute, Ulster University, Coleraine BT52 1SA, Northern Ireland, UK; 2 Department of Clinical Sciences and Nutrition, Faculty of Medicine, Dentistry and Life Sciences, University of Chester, Chester CH1 4BJ, UK

**Keywords:** Manuka honey, infection, bacteria, antibacterial, UMF, methylglyoxal, bacterial resistance

## Abstract

The importance of honey for medicinal purposes is well documented in some of the world's oldest literature. Honey is well known and studied for its antimicrobial properties. The medicinal properties in honey originate from the floral source used by bees. Manuka honey is a dark monofloral honey rich in phenolic content, and currently it is gaining much attention for its antimicrobial activity. Researchers have found that honey is effective against a wide range of pathogens. The antibacterial potency of Manuka honey was found to be related to the Unique Manuka Factor (UMF) rating, which is correlated with the methylglyoxal and total phenols content. It is reported that different types of Manuka honey have differing effects and Gram-negative bacteria are more resistant than Gram-positive bacteria. Bacterial resistance to honey as antimicrobial agent has yet to be identified, possibly due to the presence of a complex mixture of methylglyoxal and other components. Honey is also reported to alter a bacterium's shape and size through septal ring alteration, which affects cell morphology and growth. Research has shown that Manuka honey of different UMF values has medicinal properties of interest and it can be beneficial when used as a combination treatment with other antimicrobial agents.

## 1. Introduction

This article deals with the medicinal properties, mainly antibacterial activities, of Manuka honey and the information included here is based on the research published on this subject. Some of the medicinal properties of plants can be constituted in their nectar, therefore, honey from different origins possess different properties. Generally, honey is used as a food ingredient, rich in specific properties. Manuka honey is a monofloral honey, which has been recently researched in many scientific labs for its unique properties, mainly for its antimicrobial activity. The reason to study antibacterial property of honey is to find safe and natural antibiotics; since several microorganisms have developed resistance to commonly prescribed antibiotics, there is a need to find alternatives. Several strains of microorganisms, as pathogens are abundant in our surrounding environment with various rates of growth and resistance mechanisms. As a result, several infections and many different diseases can develop. The pathogenicity of microorganisms can be dependent on the organisms' unique virulence factors, and the risk factors presented by the host, such as an immune-compromised system of host, and usage of intravenous drugs on regular basis [1].

Pharmaceutical companies have commercially produced a wide range of antibiotics, and one particular type of drug is prescribed by medical practitioners to patients as a specific antibiotic to treat infections caused by a particular group of bacteria. The action of antibiotics on different species of bacteria is not same due to the composition of bacterial cell envelope. Bacterial strains are categorized either as Gram-positive, or Gram-negative. The distinction between, Gram-positive and Gram-negative bacteria is due to the fact the Gram-positive bacteria react positively to Gram stain (dye), which binds to their (thick) peptidoglycan wall. By comparison, the Gram-negative bacteria, which have a thinner peptidoglycan wall do not retain Gram dye, as this washes readily away when distained using alcohol. Gram-negative bacteria possess the 3-layered cell-envelope, consisting of a thin peptidoglycan layer positioned between an outer membrane (OM) and inner bi-lipid membrane. The asymmetric OM with outward facing lipopolysaccharide-rich layer, offers extra protection from lysozyme and other agents.

Gram-positive strains of bacteria can be tolerant to extreme conditions, as they have capability of withstanding high concentrations of salt and osmotic pressure, owing to the rigid peptidoglycan layer [2]. Gram-negative bacteria tend to exhibit high drug resistance owing to the action of the OM missing in the Gram-positive bacteria. The effectiveness of antibiotics on different species of bacteria is not the same due to diverse resistance mechanisms. The OM and the generally more complex 3-layered cell-envelope of Gram-negative bacterial accounts for increased resistance to some antibiotics. Other resistance mechanism includes the formation of extracellular biofilms, which present physical barriers to antibiotic. Destruction of the antibiotic drug by enzymatic modification and/or the modification of the drug target-site, are other mechanisms for resistance. Multi-drug resistance is the result of bacteria expelling unrelated antibiotic agents using efflux transporters, which operate synergistically with the OM to confer protection of Gram-negative bacteria [2],[3]. In recent years due to over-usage of prescribed antibiotics, microorganisms have developed resistance to many antibiotics. There is a need of an alternative therapeutic agent. In this review, we have focused on one such natural material, Manuka honey, which could be used as a natural antibiotic and as an alternative medicine.

## 2. Bacterial infections

Gram-negative organisms were reported to be more pathogenic due to their outer membrane in comparison to Gram-positive organisms, as described above. *Pseudomonas aeruginosa* is a hospital-derived pathogen and it is evidently known for its antibiotic resistance. It is an opportunistic organism and can cause serious infections in a patient suffering with other illnesses, most commonly cystic fibrosis. Some Gram-positive organisms such as members of *Staphylococcus* genus have been known as a common leading cause of bacterial infections [3]. These Gram-positive organisms successfully colonise the skin and mucosal membranes of the host. *Staphylococcus* bacteria can be transmitted through skin-to-skin contact or exposure via inhalation of infectious particles; *S.* *aureus* is a commensal bacterium and pathogen in humans with approximately 30% of the population colonised with this bacterium [4].

Tissue-specific infections: The infections in skin, soft tissue and surgical sites are commonly caused by *S.* *aureus* [5]. *S.* *epidermidis* is another common member of the *Staphylococcus* genus found within microbiota of the human skin. *S. epidermidis* is a Gram-positive pathogen, and is one of over 40 species belonging to the genus *Staphylococcus* [6]. Common occurrence within the microbiota of the host has resulted in a mutual relationship with a possibility of providing probiotic function. As an opportunistic pathogen, it is a frequent source of nosocomial and common infections on medical equipment due to its common occurrence on the skin microbiota [7]. Though it is a rare cause of fatal disease but can be costly and difficult to treat. In the United States, treatment costs were approximately $2 billion annually, due to treatment complications such as resistance genes to antibiotics and biofilms formation. In recent studies, it has been found to have specific molecular determinants, which enable *S.* *epidermidis* to evade the immune system and cause more serious diseases [8]. Manuka honey may help in the treatment of such infections, such as healing diabetic ulcers. Topical applications of Manuka honey is used in the treatment of burns, ulcers and non-healing wounds. It has also been shown to combat antibiotic-resistant strains of infections, such as MRSA (Methicillin Resistant *Staphylococcus aureus*). Manuka honey was approved as a recommended alternative and a natural material for the treatment of wounds by the U.S. Federal Drug Administration in 2007. Manuka honey amongst other types of honey has its ability to release hydrogen peroxide that is an important factor for helping reduce and eliminate bacterial activity [8].

## 3. Honey's use in alternative therapy

Much research is being focused on alternative approaches to treat bacterial infections. Honey was found to have several benefits as an alternative medicine, such as in wound healing and as an anticancer agent [9]. Honey has been used as a natural medicine for more than 2000 years, mainly for wound healing. Though there are many varieties of honey, only some of them e.g. Manuka honey and Malaysian Tualang honey, have been studied in detail for their medicinal properties. Evidence from clinical trials, shows that honey may be useful for treatment of damage to the epithelial barriers due to burns injury [10]. Honey contains medicinal agents originating from plant nectar. The antimicrobial properties are from the honey's ability to inhibit bacterial growth, which have been demonstrated using many microorganisms, including *S. aureus*, *S. pyogenes*, *P. aeruginosa* and *E. coli* [9],[10].

### 3.1. Unique Manuka Factor honey

Manuka honey is a monofloral honey, produced from the nectar of flowers of Manuka tree. This variety is produced from the *Apis mellifera* honey bees, using New Zealand Manuka plants producing specific floral-variety named as *Leptospermum scoparium* [11]. Manuka honey is usually rated using a classification system known as the Unique Manuka Factor (UMF), which reflects the equivalent concentration of phenol (%, w/v) required to produce the same antibacterial activity as honey.

The composition of Manuka honey consists of carbohydrates, minerals, proteins, fatty acids, phenolic and flavonoid compounds. Although such compounds are found in other types of honey, other unique features also occur in Manuka honey, such as an unusually high level of methylglyoxal (MGO) formed from dihydroxyacetone (DHA) which correlates with antibacterial activity [12]–[14]. Kato et al. also noted the occurrence of methyl syringate glycoside (leptosperin) as a unique maker for Manuka honey authentication [15]. Interestingly, the UMF rating of Manuka honey strongly correlates with MGO equivalence and antibacterial activity but the relation is not wholly understood [16].

In addition to antibacterial activity [11]–[16], UMF honey has the ability to stimulate macrophages through Apalbumin 1 protein to release mediators such as TNF-α, IL-1β and IL-6, which are needed for reducing microbial infections and helping in tissue healing [17].

Manuka honey shows antioxidant and anticancer properties, which are considered due to its constituents-phytochemicals working as active bio-compounds [18]–[20]. A detailed *in vitro* study reported that the total phenol content and the antioxidant activity of Manuka honey influences the cytotoxic effects on MCF-7 cells [13]. Kwok et al. used a rapid colorimetric assay to monitor MGO content and other dicarbonyl compounds from Manuka honey as a cheaper alternative method to HPLC analysis. It was found that the MGO levels of Manuka honey are 20-fold higher in comparison to other non-Manuka honeys, but currently the precision of this method is quite low [21]. The recently published research has also concluded that the UMF rating for Manuka honey correlates with its antioxidant capacity and with the total phenol content analysed in honey of all grading of UMF [22].

### 3.2. Antibacterial activity of Manuka honey

#### 3.2.1. Effect of honey on bacterial growth and cell morphology

Honey's antimicrobial activity has been attributed to its MGO and hydrogen peroxide content [9],[10]. Other factors such as osmotic pressure, pH, low protein content, bee defensin-1, the hyper-osmolality effect, different levels of flavonoids and phenolic complexes, as well as its high carbon to nitrogen ratio have also been taken into consideration for contributing to its activity [23]. There are publications from recent studies, which have indicated the antibacterial activity of honey in terms of minimum inhibitory concentration (MIC), that is the minimum concentration of honey required to inhibit the microbial growth [20],[21]. Manuka honey has proved the front-runner of honeys for non-peroxide antimicrobial activity. Tonks et al. have reported that Manuka honey's antibacterial activity is independent in inducing inflammatory cytokines during an innate immune response [24]. They identified a component, which is heat-sensitive, protease-resistant and did not express antibacterial activity but can induce cytokine production through interacting with TLR4 on macrophages. Depending on the bacterial species, Manuka honey can alter a bacterium's shape and size such as affecting the septal ring, which mediates the cell division [25].

Henriquez et al. studied *S. aureus* cultures using transmission electron microscopy, which were treated with Manuka honey, and from this study they reported that the samples had more cells with completed septa compared to cells treated with artificial honey [26]. Even though the septa were complete, it was suggested that the septa might have formed prematurely and cell division was interrupted. This was concluded that though the cells failed during cell division but appeared as normal when observed using scanning electron microscopy. Lu et al. used phase-contrast imaging on cells of Bacillus subtilis and S. aureus, which were exposed to Manuka honey at a sub-lethal dose (4%, w/v). Both species produced smaller cells and condensed chromosomes, which was identified through DAPI staining [24]. Even though the studies carried out by Henriquez et al., [26] and Lu et al. [25] used different honey treatments carried out at different times, but interestingly both studies showed that the stresses exerted by the honey on the bacteria's environmental and nutritional requirements caused unregulated growth and also affected the cell division in bacteria.

#### 3.2.2. Effect of honey on biofilms

Lu et al. [27] quantified cell viability of *S. aureus* in biofilm after their treatment with honey using BacTitre Glo Microbial Cell Viability Assay Kit, which measures cell viability through ATP levels. Manuka-type honeys may oppose biofilm production at low concentrations, which was proposed to be induced by a stress-response in the same manner that an antibiotic would exert. Reduced biofilm formation was influenced by MGO and the sugar content of the honeys, but this was not the sole influence of anti-biofilm activity. On closer examination of the biofilm, honey was found to reduce biofilm mass by killing bacterial cells entrapped in the biofilm matrix. Moreover, planktonic cells released from the biofilm did not exhibit resistance to the honey samples. From these results, Lu et al. suggested a suitable therapeutic level of a Manuka-type honey, which could be used as a topical treatment for chronic wound infection [27]. In another report also bacterial resistance to honey was not reported, Cooper et al. has stated that this property may be due to the complexity of honey components, which work individually or in a synergistic manner to prevent the resistance to honey [28].

#### 3.2.3. Effect of factors on antibacterial property of honey

An effective application in wound healing is the use of Manuka honey derived hydrogels, which have gained interest as a preventative measure in tackling potential infections. No cytotoxic effects were observed on human mesenchymal stem cells applied to Manuka hydrogels, which inhibited the growth of *S. aureus* and *S. epidermidis* [29]. This has been studied that Manuka honey of different Unique Manuka Factor (UMF) show selective activity against different bacteria. The UMF value refers to the methylglyoxal (MGO) content of the honey and it is suggested that this is responsible for much of the honey's antibacterial properties. In a recent study Almasaudi et al. compared the effects of five types of honey: Manuka honey UMF 20+, Manuka honey UMF 16+, active honey 10+, Sidr honey and *Nigella sativa* honey against 2 strains of *S. aureus* [30]. The inhibition of bacterial growth by all types of honey was evident at 20% and 10% concentrations (v/v). Researchers have found that Manuka honey had a higher antibacterial activity against both strains, with a stronger effect coming from the honey with higher UMF. It was suggested that the reason for their antibacterial potency comes from the fact they have a higher total number of phenols, which have an ability to scavenge superoxide free radicals [30],[31]. Although the polyphenols may not be a major influence in antibacterial activity and from previous finding they could not contribute entirely to antibacterial activity, or potentially the concentration of phenolic compounds were too low to exert an antimicrobial effect.

There is significant evidence for a correlation between MGO concentration and antibacterial activity, whereas there is evidence of slight antibacterial activity in the high sugar content and acidity [12]–[16]. Researcher has discussed the contributing factors, such as the source of nectar, geographical-location of flowers and the weather, as well as the storage period and conditions; therefore, the tested samples were stored in bottles in the dark and under cold conditions [11]. The findings from this study coincide with findings from reports where honey was used against antibiotic-resistant bacteria in burn wounds [31]. Report concluded that the MGO or the non-peroxide activity may be the source/cause of the honey's antibacterial activity, however, the exact compound, which is responsible is still to be confirmed. In a recent study, researchers tested three specific chemical markers (methyl glyoxal content, methyl syringate and phenyllactic acid) and found in the disk diffusion assay that MGO exerted antibacterial activity, while no activity was recorded by methyl syringate against *E. coli*, *S. aureus*, and *B. subtilis* strains [32],[33]. An interesting point was noted that how differently Gram-negative and Gram-positive bacteria reacted to honey [34]. In a study carried out, the researchers compared the antibacterial effect of Manuka honey against Enterococcus faecalis and E. coli, a Gram-positive and a Gram-negative bacterium. The results from this study indicated that Gram-negative bacteria might be more resistant when exposed to Manuka honey [35].

#### 3.2.4. Honey's use for resistant bacteria and biofilms

Paramasivan et al. [36] tested the MGO content of Manuka honey against biofilm through an *in vivo* model (ovine frontal sinuses); due to the reason bacterial biofilms increase the resistance in chronic rhinosinusitis (CRS) patients. They tested MGO alone and in combination with Manuka honey (≤1.8 mg/mL). Sinus irrigations were performed with Manuka honey/MGO concentrations between 0.9 and 1.8 mg/mL [36]. Schneider et al., tested the antimicrobial activity of a Scottish honey (Portobello) produced from the Portobello orchard in Edinburgh and Manuka honey [37]. Two methods were used to test the activity: broth dilution assay and agar disk diffusion method. The broth dilution assay showed antimicrobial activity from Manuka and Portobello, inhibiting most *S. aureus*, *P. aeruginosa* and *E. coli* cultures at concentrations 50% and 70%. Schneider et al. did not calculate MIC because honey did not diffuse properly and uniformly onto the agar from the discs, this was thought due to the use of raw honey in test instead of using honey extract [37].

Paramasivan et al. reported that there had been no resistant bacteria isolated from those wounds, which had been exposed to sub-inhibitory concentrations of Manuka honey [36]; this was anticipated to be likely due to the differences in honey, such as the levels of two main antibacterial components, hydrogen peroxide and MGO, depending on their geographic locations and floral sources. The differences in honeys' phenolic profiles can be influenced by their floral origin. This includes the diversity of floral resources in multi-floral honeys and single-origin honeys, which may be derived of floral sources with higher or lower phenolic content. For example, researchers found that the total phenolic content depended on the hive location, as the total phenolic content was found higher in urban multi-floral honeys compared to rural multi-floral honeys. Thus, it was concluded that it is not solely influenced by one specific species but by the floral resource diversity [36]. The effectiveness of honey as an antibacterial agent was found to increase as the level of MGO increased in honey, but other samples with the same level of MGO not containing sugar were unable to prevent biofilm formation. From this study they concluded that there must be other preventative factors against biofilm formation.

Lu et al. [25],[27] tested Manuka (M), Kanuka (K), Manuka-Kanuka (MK) types of honey from different floral sources against bacterial cultures. The authors reported that some of their observations did not fit in the trend in terms of MGO and hydrogen peroxide concentration influence. Instead, each honey exhibited different effects on each bacterium. For instance, one of the Manuka honeys MK1 had the lowest MGO level but had the highest antibacterial activity against *B. subtilis*, *E. coli* and *S. aureus*, when exposed to catalase. MK2 showed a sharp drop in its inhibitory activity for *B. subtilis* to 16%, when exposed to catalase. MK3 showed partial inhibition of *P. aeruginosa*, which was only evident, when it was not exposed to catalase. Lu et al. concluded from this work that the presence of MGO and hydrogen peroxide did not solely contribute to the inhibition of bacterial growth [25],[27], as Alvarez-Suarez et al., had stated there must be other components, which perform an independent action or aid in exerting MGO and hydrogen toxicity [17].

#### 3.2.5. Synergistic activities using honey with conventional-antibiotics

*In vitro* studies suggest that honey can reverse antibiotic resistance. Therefore honey could be used as part of a combination treatment for resistant-bacterial infections. For example, researchers reported synergy between oxacillin and Manuka honey sensitizing methicillin-resistant *Staphylococcus aureus* to oxacillin [38]. Müller et al. used Manuka honey in combination with the antibiotic rifampicin and found that this combination was able to inhibit MRSA in clinical isolates. The reversal in antibiotic resistance was explained as due to honey inducing a down-regulation of *mecR1*, which is a resistance gene in MRSA [39].

Synergistic activities of antibiotics and Manuka honey is an interesting area of research. As described earlier Manuka honey has been proven to have anti-biofilm activity [27],[36], therefore, the researchers experimented combining Manuka with conventional antibiotics for the treatment of such chronic infections caused by the biofilms [40]. Medihoney, which is the global leading line of medical-grade honey products for the management of wounds and burns, produced from the *Leptospermum* species of Manuka plant in New Zealand, was combined with standard antibiotics. The viability assays with checkerboard micro-dilution were used in the study, researchers have reported that the Rifampicin combination was the most effective against staphylococcal biofilms [40]. The biomass of biofilm and viability of *S. aureus* cells were significantly reduced. Interestingly, some antibiotic-honey combinations proved to be antagonistic (Gentamicin and Oxacillin), and other combinations, such as Fusidic acid and Clindamycin proved to be synergistic and antagonistic, dependent upon concentration. The results of this study [39],[40] were shown to be similar to that of previous studies, which had investigated the planktonic state and biofilm formation [27],[36]. Oxacillin, Clindamycin and Rifampicin honey combinations exhibited synergy, whereas the Gentamicin-honey combination had no synergy or antagonism. These findings differ from that of the 2017 study by Liu et al., however, the Rifampicin-honey combination has a very clear consistency [40]. This review does not include any information on clinical studies performed on human patients.

## 4. Conclusions

The review of selected published work provides a conclusive information that the potential importance of honey for medicinal purposes cannot be underestimated. The research data has confirmed that Manuka honey's antibacterial activity, in comparison to non-Manuka honey, is due to a higher phenolic and methylglyoxal content. Manuka honey can be safely used as an alternative natural antibiotic, which exerts a stimulating effect on macrophages to release mediators needed for tissue healing and reducing microbial infections. Unique Manuka Factor (UMF), which depends on methylglyoxal content is also important for honey's antibacterial activity. Other active components include hydrogen peroxide, acidic pH level, hyper-osmolality effect and bee defensin-1 etc. Finally, the conclusion is that honey is a natural and safe antibiotic, since no literature published has reported bacterial resistance for honey, which is attributed to the complexity of honey components working solely or in a synergistic manner with other components.

Therefore, based on above conclusion it is an interesting aspect for further research that the synergistic combination of Manuka honey of different UMF values with commercial antibiotics could be studied to establish an alternative approach for the treatment of antibiotic-resistant microorganisms.
